# Ion Transporters and Abiotic Stress Tolerance in Plants

**DOI:** 10.5402/2012/927436

**Published:** 2012-06-03

**Authors:** Faïçal Brini, Khaled Masmoudi

**Affiliations:** Plant Protection and Improvement Laboratory, Centre of Biotechnology of Sfax (CBS), University of Sfax, P.O. Box 1177, 3018 Sfax, Tunisia

## Abstract

Adaptation of plants to salt stress requires cellular ion homeostasis involving net intracellular Na^+^ and Cl^−^ uptake and subsequent vacuolar compartmentalization without toxic ion accumulation in the cytosol. Sodium ions can enter the cell through several low- and high-affinity K^+^ carriers. Some members of the *HKT* family function as sodium transporter and contribute to Na^+^ removal from the ascending xylem sap and recirculation from the leaves to the roots via the phloem vasculature. Na^+^ sequestration into the vacuole depends on expression and activity of Na^+^/H^+^ antiporter that is driven by electrochemical gradient of protons generated by the vacuolar H^+^-ATPase and the H^+^-pyrophosphatase. Sodium extrusion at the root-soil interface is presumed to be of critical importance for the salt tolerance. Thus, a very rapid efflux of Na^+^ from roots must occur to control net rates of influx. The Na^+^/H^+^ antiporter *SOS1* localized to the plasma membrane is the only Na^+^ efflux protein from plants characterized so far. In this paper, we analyze available data related to ion transporters and plant abiotic stress responses in order to enhance our understanding about how salinity and other abiotic stresses affect the most fundamental processes of cellular function which have a substantial impact on plant growth development.

## 1. Introduction

Agricultural productivity is severely affected by soil salinity. Environmental stress due to salinity is one of the most serious factors limiting the productivity of agricultural crops, most of which are sensitive to the presence of high concentrations of salts in the soil. There are two main components to salinity stress in plants; an initial osmotic stress and a subsequent accumulation of toxic ions which negatively affects cellular metabolism [1]. In addition, it can lead to secondary stresses such as nutritional imbalance and oxidative stress [2]. The Na^+^ cation is chaotropic and predominantly associated with the deleterious effect of salinity, and therefore, most research has focused on this mineral. However, plant adaptation to salt stress also requires appropriate regulation of Cl^−^ homeostasis [3]. Indeed, for species such as soybean, citrus, and grapevine where Na^+^ is predominantly retained in the roots and stems, Cl^−^ is considered more toxic since this ion is accumulated to high levels in shoot tissues, negatively impacting on essential processes such as photosynthesis. The osmotic component of salinity is caused by excess inorganic ions such as Na^+^ and Cl^−^ in the environment that decrease the osmotic potential of the soil solution and hence water uptake by the plant root. Uptake of abundantly available Na^+^ and Cl^−^ therefore, offers a comparatively cheap way to lower the tissue-osmotic potential. To avoid the risk of ion toxicity associated with this strategy, Na^+^ and Cl^−^ are generally compartmentalized in the vacuole and/or in less sensitive tissues. In parallel, adjustment of the cytoplasmic compartment is achieved via production of compatible osmolytes such as, proline, mannitol, sorbitol, and glycine betaine. The latter also acts as antioxidant and thus detoxifies reactive oxygen species (ROS). However, when plants are growing in high salt concentrations, an adequate sequestration of ions in the vacuole can become a limiting factor, especially in the case of glycophytes. In this scenario, plants can accumulate excessive amount of Na^+^ in the cytosol which negatively affects many aspects of cellular physiology. The most abundant inorganic cation in the cytosol is K^+^, in plant as in animal cells. This might be due to the fact that this cation is less chaotropic than Na^+^, that is, more compatible with protein structure even at high concentrations. The physicochemical similarities between Na^+^ and K^+^ lead to a competition at transport and catalytic sites that normally bind the essential cation K^+^ and maintaining a high cytosolic K^+^/Na^+^ ratio is believed to improve salt tolerance [4, 5]. Oxidative stress is another aspect of salinity stress which is in fact a consequence of salinity-induced osmotic and/or ionic stress [6]. The salt-induced production of ROS such as superoxide radicals (O^2−^), hydrogen peroxide (H_2_O_2_), and hydroxyl radicals (OH) has then a severe effect on cellular structure and metabolism negatively [7].

Although considerable progress was made to increase and secure crop yield through conventional breeding, the goal of improving the resistance of crops to abiotic stresses has seen limited success because of the complex, multigenic nature of the traits, and the narrow genetic variation in the gene pools of major crops. Numerous genes and proteins have been shown to affect the tolerance to environmental stress in an array of plant species, which together compose a complex puzzle with a myriad of individual elements and crisscrossing signal transduction pathways. A common theme of tolerance is the adequate control of salt uptake at the root level, regulation of influx into cells, control over long distance transport, and the compartmentation at both cellular and tissue levels [8, 9]. These processes are mediated by membrane transporters and manipulating the activity of this class of proteins has therefore enormous potential to affect plant performance in saline conditions [10]. Different approaches have been used to identify membrane transporters with putative functions in salt tolerance. Yeast is widely used as host for heterologous expression of plant proteins. Yeast complementation screens led to the isolation of plant transporters such as the vacuolar Na^+^/H^+^ antiporter *AtNHX1* [11] and the plasma membrane K^+^/Na^+^ symporter *TaHKT1* [12]. Loss of function mutants in the model system *Arabidopsis thaliana* helped characterize many membrane transporters including *AtHKT1:1* involved in long distance Na^+^ transport [13], the plasma membrane Na^+^/H^+^ antiporter *SOS1* [14], and the vacuolar pyrophosphatase *AtVP1* [15]. It is well established that uptake, efflux, translocation, and compartmentation of toxic ions (mainly Na^+^ and Cl^−^) provide important bases for salinity tolerance in plants, and hence, a potential avenue to improve crops. However, a lack of understanding regarding the molecular entities and complex interactions of the responsible membrane transport proteins has hindered progress in this respect. The present paper focuses on the main ionic constituents of salinity, Na^+^ and Cl^−^ and also analyses which specific membrane transporters have been shown, or are believed, to be involved in uptake, extrusion, long-distance transport, and compartmentalization of salt at the cellular and tissue level. Subsequently, the paper critically evaluates the reported data to assess which proteins may be particularly suitable as engineering targets to improve crop salt tolerance.

## 2. Sodium Uptake from Soil

The excess of salts in the soil solution poses a challenge to the plant. Na^+^ and other ions taken up by roots are transported to shoots in the transpiration stream, where they accumulate over time [3]. Elevated concentrations of salts are built up in the apoplast and eventually inside the cell, as water evaporates. The accumulation of ions in plant tissues results in progressive damage. These ionic specific stress effects are superimposed on those caused by hyperosmolarity [3]. Whether plants have specific transport systems for low-affinity Na^+^ uptake from soil remains an open question [16] and the exact mechanisms responsible for root Na^+^ and Cl^−^ uptake are only partially clear and likely include transporters from several gene families and transport classes.

### 2.1. The Role of Nonselective Cation Channels in Na^+^ Uptake

**Figure 1:**
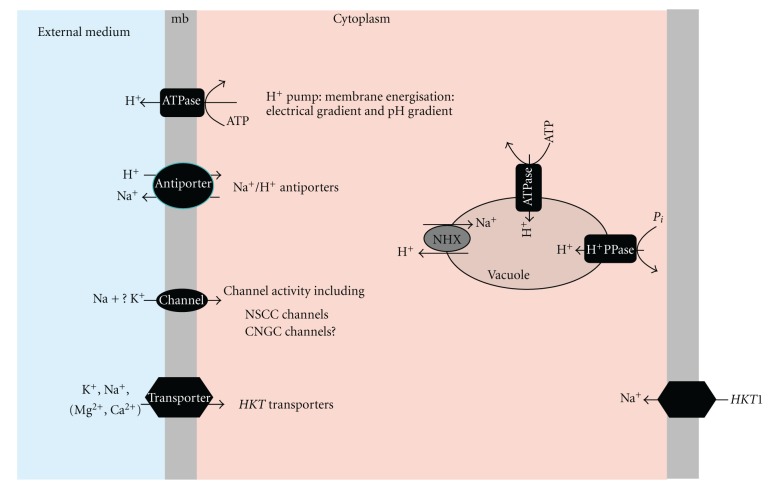
The main transport systems identified so far at the plasma membrane.

In the last few years, evidence has been presented supporting the existence of weakly voltage-dependent nonselective cation channels (*NSCC*) that are the main pathway for Na^+^ entry into the roots, at high soil NaCl concentrations [17, 18]. Although there are many candidate genes in the databases that could encode these *NSCC* channels, their identity remains elusive. Two families of nonselective cation channels, *CNGCs* (cyclic nucleotide-gated channels) [19], and *GLRs* (glutamate-activated channels) [20] have been suggested to be candidate *NSCC* channels (Figure 1) [17]. The inhibition of Na^+^ influx and *NSCC* currents upon addition of membrane permeable cyclic nucleotide analogues provided correlative evidence for the operation of *CNGCs* in plants [21], a family of plant channels that in *Arabidopsis* comprise 20 members [22]. To date, five *AtCNGCs* have been characterized (*AtCNGC1, 2, 3, 4,* and *10*) [19, 23–25]. Electrophysiological studies have suggested that *AtCNGC1* and *AtCNGC4* are equally permeable to K^+^ and Na^+^ and when expressed in *Xenopus oocytes*, they displayed activation by cyclic nucleotides [19, 23]. *AtCNGC2* appears to be selective for K^+^ and to discriminate against Na^+^ [19]. *AtCNGC10* rescued K^+^ transport defective mutants of *E. coli*, yeast, and *Arabidopsis akt1-1*, suggesting that *AtCNGC10* mediates the transport of K^+^ into the roots [24]. *AtCNGC3* was recently characterized by functional complementation of yeast and by characterization of *Arabidopsis* T-DNA knockout mutants [25]. *AtCNGC3* was primarily expressed in the cortical and epidermal root cells. Growth of the mutant seedlings in toxic NaCl (and KCl) concentrations was improved, suggesting a restricted ion influx in the mutant plants [25]. Ionotropic glutamate receptors (*GLRs*) are proteins that interact with glutamate and form cation channels with a wide range of permeability's. In *Arabidopsis,* the family of putative *GLRs* comprises 20 members [20]. Glutamate-activated Na^+^ and Ca^2+^ voltage-independent currents were characterized in *Arabidopsis* roots. Demidchik et al. [20] noted that although the effects of environmental factors on apoplastic glutamate remain unclear, the concentrations of glutamate required for half activation of these channels correlated well with the range of apoplastic glutamate concentrations reported (0.2–0.5 mM), suggesting a role of these channels in Na^+^ uptake.

### 2.2. Potassium Channels May Contribute to Na^+^ Uptake

Sodium has a strong inhibitory effect on K^+^ uptake by cells, presumably by interfering with transporters in the root plasma membrane such as the Shaker type K^+^ channels (*KAT1* and *AKT1* form the predominant inward K^+^ conductance observed in plant plasma membranes). Such channels generally have a high K^+^/Na^+^ selectivity and were generally regarded not to play a significant role in Na^+^ [26, 27]. However, a more recent work suggests that the picture is more complex and there may be ecophysiological variations in this respect. Wang et al. [28] used apharmacological approach to characterize Na^+^ uptake in the halophyte *Suaeda maritima* and concluded that the low-affinity Na^+^ uptake pathway in this species resembles an *AKT1* channel. Similarly, Kader and Lindbergh [29] provide evidence that K^+^ channels mediate substantial Na^+^ influx in a salt-sensitive rice cultivar but not in a tolerant one. In both cases the conclusions were derived from applying channel blockers and inhibitors which can be notoriously nonspecific, but these findings do suggest that K^+^ channels are potential pathways for root Na^+^ influx. In addition, the study by Wang et al. [28] suggests that basic processes such as Na^+^ uptake may be considerably different in halophytes and such diversity could be an important contributor to salt tolerance. However, the scarcity in data from halophytes in this respect forms a large hindrance in testing this hypothesis.

### 2.3. Carrier-Type Transporters That Mediate Na^+^ Uptake

*HKTs* (high affinity potassium transporters) are carrier-type proteins that mediate Na^+^ and K^+^ transport (Figure 1) [30]. Members of the *HKT* gene family are Na^+^-specific transporters (although they were initially described as high-affinity K^+^ transporters and hence their name) that mediate either preferential Na^+^ transport or Na^+^-K^+^ symport, partly depending on whether the specific transporter has a highly conserved serine (subfamily 1) or glycine (subfamily 2) residue in the first pore loop of the protein and on the extracellular Na^+^-K^+^ ratio [31, 32]. Generally, *HKT* members of subfamily 1 have a relatively higher Na^+^-to-K^+^ selectivity than subfamily 2 *HKT* transporters. In *Arabidopsis*, loss of function of the only *HKT1;1* gene encoding a Na^+^-selective transporter caused the accumulation of Na^+^ in leaves but reduced Na^+^ concentrations in roots, with little effect on the net uptake of Na^+^ by the plant [13, 33, 34]. *AtHKT1;1* is preferentially expressed in the vasculature, where it is thought to regulate the Na^+^ distribution between roots and shoots [13, 31–35]. Two complementary functions for *AtHKT1;1* have been proposed [36]. The phloem recirculation model posits that Na^+^ is loaded into shoot phloem cells by *AtHKT1;1* and then transferred to roots via the downward stream of phloem, preventing Na^+^ over accumulation in shoots (Table 1) [37]. However, there seems to be little (10% or less) retranslocation of Na^+^ from leaves via the phloem relative to the amount imported in the transpiration stream via the xylem [17, 38, 39]. On the other hand, *AtHKT1;1* is generally accepted to mediate the retrieval of Na^+^ from the xylem sap, thereby restricting the amount of Na^+^ reaching the photosynthetic tissues [34, 37–39]. These two Na^+^ transport processes could be functionally linked to achieve basi-petal translocation of Na^+^ because ions that were unloaded by xylem parenchyma cells might be transported into the phloem *via* symplastic diffusion [36]. Engineered expression of *AtHKT1;1* in the root pericycle of *Arabidopsis* enhanced inward Na^+^-transport in the targeted cells, reduced root-to-shoot transfer of Na^+^, and improved salt tolerance [40]. However, it remains unclear whether the reduced activity of *AtHKT1;1* was the sole basis for enhanced tolerance or if there were other processes that could also contribute to salt tolerance linked to enhanced Na^+^ accumulation such as improved capacity for Na^+^ sequestration in vacuoles [41]. Similar studies in cereals have shown that natural variation in the activity or expression of *HKT* transporters may be a genetic resource for enhanced NaCl tolerance. In rice, two of the nine members, *OsHKT2;1* and *OsHKT2;2*, are expressed in roots amongst other tissues [42]. *OsHKT2;2* catalyses Na^+^-dependent K^+^ uptake [43]. *OsHKT2;1* has been shown to catalyse high-affinity Na^+^ uptake into roots under K^+^-starvation conditions, and it appears that Na^+^ can partially replace the function of K^+^ under such conditions [43]. *OsHKT2;1* has been shown to catalyse high-affinity Na^+^ uptake into roots under K^+^-starvation conditions, and it appears that Na^+^ can partially replace the function of K^+^ under such conditions [43]. Expression of *OsHKT2;1* is localized to the root epidermis, cortical cells, and vascular tissues of both roots and leaves [43–45], and expression patterns in roots were found to be different in salt-tolerant and sensitive varieties in response to NaCl stress [44]. Loss of function mutants in *OsHKT2;1* shows reduced growth in low K^+^ conditions and accumulated less Na^+^ [43]. Thus, it appears that *OsHKT2;1* augments monovalent cation uptake by providing high-affinity Na^+^ uptake in K^+^ deficient conditions. However, *OsHKT2;1* relevance in Na^+^ uptake during salinity stress may be limited since it has a micromolar affinity for Na^+^, and its activity is rapidly downregulated at higher ambient concentrations of Na^+^. Similar high-affinity Na^+^ uptake was observed in K^+^-starved barley roots. However, when heterologously expressed in yeast, *HvHKT2;1* was shown to mediate Na^+^ (or K^+^) uniport, Na^+^-K^+^ symport, or a combination of both, depending on the construct from which the transporter was expressed [30, 46]. These characteristics suggest that the *HKT* transporters are potentially of importance in the regulation of Na^+^ influx into roots. Several QTLs responsible for variation of K^+^ and Na^+^ content were mapped to *HKT* family genes. QTLs analyses showed that greater shoot K^+^ content of the relatively salt-tolerant rice cultivar Nona Bokra cosegregated with the presence of an allelic variant of *SKC1* (shoot K^+^ content) with greater activity relative to that of the salt-sensitive Koshihikari variety [47]. *SKC1* (renamed *OsHKT1;5*) is a plasma membrane K^+^-independent, Na^+^-selective transporter that is preferentially expressed in the parenchyma cells surrounding xylem vessels. The greater Na^+^ concentration in the xylem sap and leaves of the salt-sensitive variety would be a consequence of a weaker *SKC1* allele and reduced Na^+^ reabsorption from the xylem. Quantitative genetic analyses in wheat have also led to the identification of two loci, *Nax1* and *Nax2*, which reduced Na^+^ accumulation in the leaf blade by excluding Na^+^ from the xylem by two different mechanisms [38]. The process controlled by *Nax2* was confined to the roots and had the effect of reducing the transport of Na^+^ from root to shoot, presumably by improved discrimination between Na^+^ and K^+^ at the point of xylem loading. The *Nax1* locus enhanced the retention of Na^+^ in the leaf sheath, thus restricting further passage to the leaf blade [38]. High-resolution mapping and sequencing analyses of known Na^+^ transporter genes have suggested that the *Nax1* and *Nax2* loci are attributable to polymorphisms in wheat *HKT* genes encoding proteins of the subfamily 1 with preferred Na^+^ transport [48, 49]. These results strongly indicate that Na^+^ exclusion from the transpiration stream may be an important mechanism in the salt tolerance of cereals, similar to many other plant species [17]. It should be pointed out, however, that most studies concerning QTL analysis for salt tolerance are based in Na^+^ and/or K^+^ content in tissues or organs and not directly in salt tolerance. Often, higher Na^+^/K^+^ ratios are regarded as determinants of salt tolerance itself without considering any other agronomical or physiological traits. In fact, the *SKC* QTL of rice did not show a significant correlation coefficient with survival to salt stress [50]. A clear difference should be made between QTLs responsible of ionic balance and QTLs for salt tolerance. In addition to *HKTs*, other carriers have been implicated in Na^+^ uptake. Some members of the high-affinity K^+^ uptake transporter family *HAK/KUP/KT* may transport Na^+^ with low affinity in the presence of high Na^+^ : K^+^ ratios [51]. Furthermore, yeast expression studies revealed that the normal function of *HAK/KUP/KTs*, high-affinity K^+^ uptake, is competitively inhibited by Na^+^, pointing to a shared transport pathway of the two monovalent cations [52, 53]. Several studies have shown substantial transcriptional regulation of *HAK/KUP/KT* isoforms by salt stress [54–56]. For example, Su et al. [57] observed that the expression of *HAKs* in *Mesembryanthemum crystallinum* was upregulated during salt stress and K^+^-starved conditions. However, whether this result and those for other *HAKs* relate to a potential role in Na^+^ uptake or augmentation of K^+^ uptake during salinity stress remains to be established. The low-affinity cation transporter *LCT1* from wheat functions as nonselective cation carrier conducting K^+^, Rb^+^, Na^+^, and Ca^2+^ transport in yeast [58, 59]. Expression of *LCT1* in yeast leads to an increase in cation influx and hypersensitivity to Na^+^ [60]. Addition of external Ca^2+^ was found to reduce Na^+^ influx and sensitivity, but the cation profile of the influx caused by *LCT1* resembled that of endogenous ion transport in yeast, suggesting that *LCT1* might be stimulating the ion transporters already present [60].

### 2.4. Transporters Involved in Cl^−^ Uptake

Chloride (Cl^−^) is a major osmotically active solute in the vacuole involved in both turgor and osmoregulation processes [61], with implications for the proper development of plants [62]. Despite its importance in plant biology, Cl^−^ is one of the less studied essential nutrients at the physiological and molecular levels. In contrast to Na^+^, Cl^−^ uptake in most conditions must be energized, but although there is a substantial amount of information regarding K^+^ and Na^+^ transport in plants, very little is clear about the molecular mechanisms behind the substantial Cl^−^ influx that results from salinization [9]. Genes and proteins involved in Cl^−^ transport have been very poorly studied in plants. The attention has been mainly focused on the voltage-dependent Cl^−^ channel *CLC* family [63–65]. Phylogenetic and functional analyses have shown that plant *CLC* genes encode anion channels and active Cl^−^/H^+^ antiporters localized in endosomal compartments, which are involved in NO^3−^ compartmentalization [66] and pH regulation in the *trans*-Golgi system [67]. Although the transcript abundance of several *CLCs* is affected by salinity [68], they are unlikely to contribute to root Cl^−^ uptake: Firstly, plant *CLCs* have only been detected at endomembranes which appear to exclude a role in Cl^−^ uptake and secondly the thermodynamics of Cl^−^ uptake role out passive-channel-type mechanisms. A second class of potential Cl^−^ transporters is formed by the cation chloride cotransporters (CCCs) encoding one gene in *Arabidopsis* and two genes in rice. The *Arabidopsis thaliana* cation-Cl^−^ cotransporters (*AtCCCs*), expressed in root and shoot tissues, mediate the coordinated symport of K^+^, Na^+^, and Cl^−^ and have been postulated to participate in the long-distance transport of Cl^−^ [62]. Loss of function of *AtCCC* in *Arabidopsis* led to a changed root : shoot Cl^−^ ratio but also to a large increase in net Cl^−^ uptake arguing against a role of *AtCCC* in the uptake of this ion [62]. More recently, the *Arabidopsis* slow anion channel associated 1 (At*SLAC1*) gene was shown to encode for the guard cell plasma membrane S-type anion channel involved in stomatal closure [69, 70]. Another member of this family, At*SLAH1*, is expressed in the root vasculature suggesting a potential involvement in the long-distant transport of anions [69].

## 3. Transporters Involved in Salt Efflux

It is essential that plants possess adequate efflux systems to remove potentially dangerous ions such as Na^+^ from the cytosol. Inevitably, the mechanisms to extrude Na^+^ into the apoplast or vacuole have to be energized which typically occurs via H^+^-coupled antiport [5, 71], whereas those for Cl^−^ may be (partially) passive. Early studies on tonoplast antiporters showed significant upregulation of their pumping capacity after plant exposure to salt [72, 73]. In the plasma membrane too, evidence for H^+^ : Na^+^ antiporters was obtained underlining the relevance of such systems to plant salt tolerance [74]. Data dealing with Cl^−^ efflux are scarce: using compartmental flux analysis, Britto and Kronzucker [75] showed large Cl^−^ efflux when plants were exposed to 100 mM NaCl. Just as is the case for Na^+^, the majority (up to 90%) of Cl^−^ that entered the symplast was quickly removed. Although some of the Cl^−^ efflux could theoretically be mediated by anion channels, no data are available regarding the mechanistic details or regarding the identity of the proteins involved.

### 3.1. Na^+^ Efflux Mechanisms at the Plasma Membrane

Comparisons of unidirectional Na^+^ fluxes and rates of net accumulation of Na^+^ in roots indicate that 70–95% of the Na^+^ fluxed into the root symplast is extruded back to the apoplast, and that small differences in Na^+^ exclusion capacity lead to major changes in the net accumulation of Na^+^ [17]. In *Arabidopsis*, the plasma membrane Na^+^/H^+^ exchanger *SOS1* (Salt Overlay Sensitive) facilitates Na^+^ homeostasis by extruding the ion from root epidermal cells at the root-soil interface (Table 1, Figure 1) [76, 77]. *SOS1* is preferentially expressed in xylem parenchyma cells, and analyses of the Na^+^ root/shoot partitioning in roots of *sos1* plants under different salt regimes indicate that *SOS1* participates in the redistribution of Na^+^ between the root and shoot, likely working in concert with *AtHKT1;1* at the plasma membrane of xylem parenchyma cells [34, 36, 76, 78]. Additional evidence of the involvement of *SOS1* in long-distance Na^+^ transport has been produced recently in the halophytic *Arabidopsis* relative *Thellungiella salsuginea* (a.k.a. *T. halophila*) and in tomato [79, 80]. Lower net Na^+^ flux was observed in the xylem sap of tomato plants with suppressed *SOS1* activity [80]. Downregulation of *ThSOS1* in *Thellungiella* increased Na^+^ accumulation in the root tip and in the stele. Maximal Na^+^ accumulation, concomitant with a decrease in the K^+^ content, was found in the root xylem parenchyma. These cells presented a Na^+^-K^+^ ratio more than 12 times higher than equivalent cells in wild-type plants. Reduced or abolished activity of *SOS1* interferes with K^+^ nutrition and long-distance transport [80]. Mutations in rice and *Arabidopsis HKT* Na^+^ transporters also reduce K^+^ accumulation in shoots during salt exposure [34, 47]. The activity of the *SOS1* exchanger is regulated through protein phosphorylation by the *SOS2-SOS3* kinase complex in *Arabidopsis* (Table 1) [77, 81]. *SOS2/CIPK24* is a serine/threonine protein kinase of the *SnRK3/CIPK* family. *SOS3/CBL4* is a myristoylated membrane bound Ca^2+^ sensor belonging to the recovering-like family of *SCaBPs/CBLs*. Upon Ca^2+^ binding, *SOS3* binds to and enhances the protein kinase activity of *SOS2* [82]. Besides activating *SOS2*, *SOS3* was shown to recruit *SOS2* to the plasma membrane to facilitate interaction with *SOS1* [81]. *SOS2* also interacts with *SCaBP8/CBL10* to form an alternative protein kinase complex that regulates *SOS1* at the plasma membrane [83]. *SOS2* has recently been shown to phosphorylate *SCaBP8/CBL10* at its C-terminus [84], thus adding a new layer of regulation to CBL proteins besides Ca^2+^ binding and fatty acyl modifications [85]. This phosphorylation was induced by salt stress, occurred at the membrane, stabilized the *SCaBP8-SOS2* interaction, and enhanced plasma membrane Na^+^/H^+^ exchange activity [84]. Surprisingly, interaction of *SOS2/CIPK24* with *SCaBP8/CBL10* may also result in localization of the kinase complex at the vacuolar membrane where it mediates salt tolerance by regulating the accumulation of Na^+^ in shoot tissues by an as yet undefined mechanism that may involve regulation of the Na^+^/H^+^ exchange at the tonoplast [86, 87]. Regulation of the tonoplast *V-ATPase* by *SOS2* in the absence of CBL proteins has also been reported [88]. Presumably, the posttranslational modifications of *SCaBP8/CBL10* or the interaction of combinatorial protein kinase complexes with specific targets in different cellular membranes may ultimately define the localization of the protein kinase *in vivo*.

### 3.2. Sodium Compartmentation: The Vacuolar Na^+^/H^+^ Antiporter and the H^+^ Pump

The compartmentation of Na^+^ ions into vacuoles provides an efficient mechanism to avert the toxic effects of Na^+^ in the cytosol. The transport of Na^+^ into the vacuoles is mediated by cation/H^+^ antiporters that are driven by the electrochemical gradient of protons generated by the vacuolar H^+^-translocating enzymes, the H^+^ ATPases and the H^+^ pyrophosphatase (H^+^-PPase) (Table 1, Figure 1). Although the activity of these cation/H^+^ antiporters was demonstrated more than 20 years ago [72], their molecular characterization was only possible after the *Arabidopsis* genome-sequencing project. Na^+^ compartmentation in the vacuole occurs in all tissues and is an important mechanism for osmotic adjustment and Na^+^ detoxification in the cytosol. There are eight *NHX* gene family members in *Arabidopsis* [89], and the functions of *AtNHX1, 4, 7* and *8* have been studied. *AtNHX7* is also known as *AtSOS1* and *AtNHX8* has been shown to be a Li^+^/H^+^ antiporter [90], although the biological relevance of Li^+^ transport remains obscure. *AtNHX4* is localized to the vacuole and might function in concert with *AtNHX1* (Table 1) [91]. Several reports indicate that constitutive overexpression of the vacuolar transporters increases the salt tolerance of a variety or species. Constitutive overexpression of the *Arabidopsis* vacuolar Na^+^/H^+^ antiporter, *AtNHX1*, appears to increase salinity tolerance significantly in yeast [92], *Arabidopsis* [11], tomato [93], *Brassica napus* [94], and cotton [95]. Similarly, constitutive overexpression of various cereal homologs has been reported to improve the salinity tolerance of *Arabidopsis* [96], rice [97, 98], wheat [99], and barley [100]. The overexpression of *NHX1* appears to increase the capacity of the plant to regulate cytoplasmic and vacuolar pH [101, 102]. The cation selectivity is regulated by a luminal C-terminus [103]. The overexpression of *NHX1* in *Arabidopsis* led to a small increase in shoot Na^+^ accumulation [11], possibly allowing the cells to maintain a favourable osmotic balance, yet maintaining low cytoplasmic Na^+^ levels due to sequestration of the Na^+^ within the vacuole. The *nhx1* mutant had significantly lower Na^+^/H^+^ and K^+^/H^+^ exchange capabilities in isolated vacuoles, fewer large epidermal cells, and less overall leaf area, indicating that *NHX1* also plays a developmental role [104]. Overexpression and knockout of the *NHX1* gene in *Arabidopsis* have been shown to significantly and differentially alter the expression of a large number of genes involved in the response to salt stress, indicating that *Arabidopsis* can respond to a change in one Na^+^ transporter by regulating other genes [105, 106]. Other members of the *NHX* family are also capable of moving Na^+^. Yokoi et al. [89] reported that *AtNHX2* and *AtNHX5* could be important salt-tolerant determinants and observed that *AtNHX2* has a major function in vacuolar Na^+^ sequestration. H^+^ pumps in the plasma membrane and tonoplast energize solute transport necessary to compartmentalize cytotoxic ions away from the cytoplasm and to facilitate the function of ions as signal determinants [107–109]. That is these pumps provide the driving force (H^+^ electrochemical potential) for secondary active transport and function to establish membrane potential gradients that facilitate electrophoretic ion flux. The plasma membrane localized H^+^ pump is a P-type ATPase and is primarily responsible for the large pH and membrane potential gradient across this membrane [109]. A vacuolar type H^+^-ATPase and H^+^-PPase generate the ΔpH and membrane potential across the tonoplast [108, 110]. The activity of these H^+^ pumps is increased by salt treatment, and induced gene expression may account for some of the upregulation [108, 111]. The H^+^-PPases are considered to form a multigene family. Two cDNA clones (*OVP1* and *OVP2*) encoding vacuolar H^+^-PPases isolated from rice were reported [112]. Indeed, there are two genes in *Arabidopsis* annotated as inorganic pyrophosphatase H^+^-PPase (*AVP1, AVP3*) and a third loci encoding a pyrophosphatase like (*AVP2 = AVPL1*), more than five isoforms in rice, and at least three isoforms in barley [107, 113].

It has been previously demonstrated that overexpression of the H^+^-PPase *AVP1* increases salinity tolerance and Na^+^ accumulation in *Arabidopsis* [15]. The vacuolar Na^+^ levels of the transformants were found to be higher than those of wild-type plants, indicating that overexpression of *AVP1* led to increased activity of the Na^+^/H^+^ antiporter. Functional characterization of wheat Na^+^/H^+^ antiporter *TNHX1* and vacuolar pyrophosphatase *TVP1* was reported by Brini et al. [114]. Transgenic *Arabidopsis* lines overexpressing *TNHX1* or *TVP1* are much more resistant to high concentrations of NaCl and to water deprivation than the wild-type plants. These transgenic plants grow well in the presence of 200 mM NaCl and also under a water-deprivation regime, while wild-type plants exhibit chlorosis and growth inhibition [96]. In barley, expression of the H^+^-PPase *HVP1*, and the vacuolar Na^+^/H^+^ antiporter *NHX1*, was similarly upregulated by salt stress [100] and is regulated by ABA, auxin, and gibberellin [115]. The simultaneous expression of *NHX* and *AVP* genes in rice was found to increase salinity tolerance to a greater extent than expression of the genes individually [98]. The overexpression of *AVP1* also appears to increase growth rates of plants due to an interaction with the auxin transporter *PIN1*, which increases auxin transport resulting in more robust plants which are better able to survive stress conditions [24, 116].

### 3.3. Role of Cl^−^ Channels in Vacuolar Cl^−^ Compartmentation

In addition to Na^+^, Cl^−^ compartmentation is also important for salt tolerance, as elevated levels of Cl^−^ in the cytosol may be harmful, particularly in the case of citrus crops [117]. Since the vacuole is moderately positive with reference to the cytoplasm, part of the vacuolar Cl^−^ sequestration could proceed through ion channels, and several voltage-gated anion channels of the *CLC* family have been detected in the tonoplast of various species. In *Arabidopsis*, *CLCa* was recently shown to function primarily as a H^+^-coupled antiporter to drive vacuolar nitrate accumulation [66], whereas *CLCc* may also be involved in NO^3-^ homeostasis rather than vacuolar Cl^−^ sequestration. However, *CLC* transcription has been found to respond to salinity: In rice, *OsCLCa* was significantly upregulated in salt-sensitive cultivars in response to salinity stress and *OsCLCc*, which is expressed in both leaves and roots, showed transcript reduction in the chloride accumulating salt-sensitive *IR29* while transient induction occurred in the chloride excluding variety Pokkali [118]. Diédhiou and Golldack [68] showed a coordinated regulation of anion and cation homeostasis in salt-treated rice and suggested a function for *OsCLCc* in osmotic adjustment at high salinity. Nakamura et al. [119] showed that the same *CLC* channels partially complimented the yeast *gef1* mutant which lacks the yeast *CLC* channel. In conjunction, these findings suggest that *CLC*-type anion channels are important in mediating Cl^−^ sequestration in vacuole.

## 4. Long-Distance Transport of Na^+^

An important issue in salt stress physiology is Na^+^ translocation from the root to the shoot [120, 121]. Physiological evidence suggests that halophytes and salt-resistant glycophytes actively transport Na^+^ from the root to the shoot, whereas salt-sensitive glycophytes appear to limit Na^+^ entry into the transpirational stream to prevent Na^+^ accumulation in the shoot [120, 121]. The transporter(s) responsible for Na^+^ transport to and from the xylem vessels are not known, although plasma membrane Na^+^/H^+^ antiporters have been proposed to fulfil this role [111, 122]. It also is not known which cell layer(s) might be critical for controlling the extent of Na^+^ entry or exit from the xylem. The plasma membrane antiporter *SOS1* is expressed in root parenchyma and in *Arabidopsis* impacts on Na^+^ loading into the xylem sap during moderate salt stress [76]. However, its exact function may depend on the severity of the salinity stress and includes removal of Na^+^ from the xylem stream when salt stress is excessive. In *Arabidopsis*, loss-of-function mutations in the *HKT1* gene lead to overaccumulation of Na^+^ in shoots and rendered the plant Na^+^ hypersensitive [37, 123]. RNA in situ hybridizations showed that *HKT1* is expressed mainly in leaf phloem tissues and mediates Na^+^ loading into the phloem vessels. In addition, *HKT1* may be involved in Na^+^ unloading from the phloem sap in roots thus providing a mechanism for Na^+^ retranslocation from shoot to root [37]. In rice, *OsHKT1;5* is a plasma membrane Na^+^ transporter expressed in xylem parenchyma cells that retrieves Na^+^ from the xylem sap [47]. Genetic analysis revealed an important K^+^-homeostasis QTL called *SKC1* [50]. The *SKC1* gene was cloned and found to be *OsHKT1;5* [50]. Heterologous expression revealed that *OsHKT1;5* is a Na^+^ transporter, and whole plant analysis indicated that it functions in the root xylem parenchyma to retrieve Na^+^ from the xylem stream, thereby reducing Na^+^ accumulation in the shoot [50]. Flux analysis of a salt-tolerant durum wheat landrace, line 149, revealed that Na^+^ exclusion in this line is underpinned by the individual traits of decreased Na^+^ transfer to the shoot and increased Na^+^ retrieval to the leaf sheath tissue [124]. *Nax1* and *Nax2*, two previously mapped QTLs that have been linked to salinity tolerance in line149 were found to control the two transport traits [38]. The *Nax2* locus coincided with a Na^+^ transporter related to *OsHKT1;5* in rice, and this gene was shown to be responsible for removal of Na^+^ from the xylem in the roots [49]. Members of the H^+^ : monovalent cation exchanger family (*CHX*) are also likely to contribute to Na^+^ translocation. *AtCHX21* is mainly expressed in the root endodermis, and loss of function in this gene reduced levels of Na^+^ in the xylem sap without affecting phloem Na^+^ concentrations [125]. In rice, salt-induced expression of *OsCHX11* in roots was cultivar dependant and higher in a tolerant cultivar [126]. The differential expression correlated with a higher K^+^/Na^+^ ratio in the tolerant cultivar suggesting that *CHX11* may be involved in long-distance transport of Na^+^ and/or K^+^.

## 5. Engineering Plant Salinity Tolerance

Currently, a large number of potential gene targets are available to manipulate salt tolerance. This number has drastically increased through the many large scale transcriptomic studies over the past decade, but in many cases the validity of the reported findings has yet to be established. For the various processes that contribute to salt tolerance, regulating uptake of Na^+^ and Cl^−^ from the soil is of primary importance, particularly in glycophytes which appear to have unidirectional Na^+^ and Cl^−^ influx that greatly exceeds net uptake [127]. Although several studies convincingly show that nonselective cation channels are involved, their molecular nature is largely unknown. Out of the substantial gene families that encode nonselective cation channels (*CNGCs* and *GluRs*), only *CNGC3* and *CNGC10* were shown to have a moderate impact on salt tolerance [25, 128]. The data available suggest that single *CNGCs* do not play important roles in Na^+^ uptake. However, creating multiple loss of function mutants, for example, for all *CNGCs* or *GluRs* expressed at the root periphery, may be required to provide more conclusive evidence in this respect. Na^+^ efflux processes should be maximized to counteract Na^+^ influx. This might be possible by overexpressing Na^+^/H^+^ antiporters or Na^+^-ATPases specifically in the outer root cells to improve Na^+^ extrusion. The bryophyte *Physcomitrella patens* is tolerant to a range of stresses and is consequently attracting significant attention as a potential source of genes to improve stress tolerance in higher plants [129–132]. As in many algae and fungi [133], and unlike higher plants that do not have primary ATP hydrolysing Na^+^ pumps, *P. patens* has two Na^+^-ATPases: *PpENA1* and *PpENA2* [134]. Expression of *PpENA1* is significantly upregulated by Na^+^ stress, and the wild-type *Physcomitrella* maintained a higher K^+^/Na^+^ ratio and larger size than the *ena1* knockout line at moderate Na^+^concentrations (100 mM) [135]. The generation and characterization of plants with increased Na^+^ efflux from the root epidermal and cortical cells are eagerly awaited. The potential for storage of Na^+^ in vacuoles in the root should be maximized, as has been achieved by overexpression of *NHX* and vacuolar pyrophosphatase genes [11, 15]. Loading of Na^+^ into the xylem by the inner stelar cells of the root should be minimized and retrieval of Na^+^ from the xylem increased, as has been achieved through amplification of *AtHKT1;1* activity in the root stele in *Arabidopsis* [40]. Once Na^+^ has been transported to the shoot, strategies for tolerance to Na^+^ become important. These include increased storage of Na^+^ in the vacuole either through increased uptake or decreased efflux across the tonoplast. Several studies using overexpression of *NHX* and vacuolar pyrophosphatase have shown this strategy to be effective (Table 1) [11, 15, 93–96, 98–100].

## 6. Conclusion

Several abiotic stresses cause changes in morphological, physiological, biochemical, and molecular processes in plants. The increasing prevalence of soil salinity is one of the most dangerous obstacles to improving crop productivity and quality. The adverse effects of saline soil include ion toxicity, nutrient constraints, osmotic stress, and oxidative stress. Many gene targets involved in salt tolerance have been identified through various approaches, particularly through transcriptomic studies. It is likely that such approaches generate many false positives [136], and this is born out by a lack of supporting evidence for an actual function in plant salinity tolerance. The accumulative data show importance of two particular classes of transporters: *HKTs* which function in both Na^+^ uptake and long-distance translocation [47] and *NHXs* in their capacity as Na^+^ : H^+^ antiport [137] or by maintaining K^+^ homeostasis [138]. The significance of these systems is often isoform dependent and may be further complicated by allelic variation between cultivars. Manipulation of several of the genes discussed above has been shown to alter uptake, efflux, translocation, and compartmentation of Na^+^. Knowledge gained through use of heterologous expression systems and model plant systems provides an extremely useful starting point for the development of salinity-tolerant crop plants. However, further work involving the transgenic expression of Na^+^ transporters in cereal and broad leaved crop plants needs to be undertaken. Before any claims of salinity tolerance can be substantiated, robust data on yield measurements is required; preferably from field-based trials [139]. Also, it is argued that phenotyping of *Arabidopsis* should be carried out under the most relevant conditions possible, notably, under transpiring conditions rather than in sealed-agar-plate-based assays, as transpiration is a crucial factor in the transport of ions such as Na^+^ through the plant.

## Figures and Tables

**Table 1:** Plant ion transporters involved in salt tolerance identified by functional analysis.

Name	Source species	Gene product	Function	Identification method	Harbourg species	Reference
			Sodium influx			

*AtHKT1*	*A. thaliana*	Na^+^ transporter	Na^+^/K^+^ homeostasis	Mutation	*Arabidopsis*	[13]
*HKT1*	*T. aestivum*	Na^+^/K^+^ transporter	K^+^/Na^+^ homeostasis	Overexpression	Wheat	[31]

			Sodium efflux			

*AtSOS1*	*A. thaliana*	Plasma membrane Na^+^/H^+^ antiporter	Na^+^ detoxification	Mutation	*Arabidopsis* overexpression	[76]
*TaSOS1*	*T. aestivum*	Plasma membrane Na^+^/H^+^ antiporter	Na^+^ detoxification	Mutation	*Arabidopsis* overexpression	[99]

			Sodium compartmentation			

*AtNHX1*	*A. thaliana*	Vacuolar Na^+^/H^+^ antiporter	Na^+^ vacuolar sequestration	Overexpression	*Arabidopsis,*	[11]
					Cabbage, tomato	[93]
*TNHX1*	*T. aestivum*	Vacuolar Na^+^/H^+^ antiporter	Na^+^ vacuolar sequestration	Overexpression	*Arabidopsis*	[96, 114]
*AVP1*	*A. thaliana*	Vacuolar H^+^-PPase	H^+^ transport, vacuolar acidification	Overexpression	*Arabidopsis*	[15]
*TVP1*	*T. aestivum*	Vacuolar H^+^-PPase	H^+^ transport, vacuolar acidification	Overexpression	*Arabidopsis*	[96, 114]

			Regulatory genes			

*SOS2*	*A. thaliana*	Serine/threonine Protein kinase	SOS1 regulator	Mutation	*Arabidopsis*	[87]
*SOS3*	*A. thaliana*	Ca^++^ binding protein	Ca^++^ sensor/SOS2 activator	Mutation	*Arabidopsis*	[81]
